# Understanding Fossil Phytolith Preservation: The Role of Partial Dissolution in Paleoecology and Archaeology

**DOI:** 10.1371/journal.pone.0125532

**Published:** 2015-05-20

**Authors:** Dan Cabanes, Ruth Shahack-Gross

**Affiliations:** 1 ERAAUB, Department of Prehistory, Ancient History and Archaeology, University of Barcelona, c/ de Montalegre 6–8, 08001, Barcelona, Spain; 2 Kimmel Center for Archaeological Science, Weizmann Institute of Science, Rehovot, 76100, Israel; ICREA at the Universitat Autònoma de Barcelona, SPAIN

## Abstract

Opaline phytoliths are important microfossils used for paleoecological and archaeological reconstructions that are primarily based on relative ratios of specific morphotypes. Recent studies have shown that phytolith assemblages are prone to post-depositional alteration involving partial dissolution, however, the manner in which partial dissolution affects morphotype composition is poorly understood. Here we show that morphotype assemblages from four different plant species subjected to controlled partial dissolution are significantly different from the original assemblages, indicating that the stability of various morphotypes differs, mainly depending on their surface area to bulk ratios. This underlying mechanism produces distorted morphotype compositions in partially dissolved phytolith assemblages, bearing vast implications for morphotype-based paleoecological and archaeological interpretation. Together with analyses of phytolith assemblages from a variety of archaeological sites, our results establish criteria by which well-preserved phytolith assemblages can be selected for accurate paleoecological and archaeological reconstructions.

## Introduction

Silica phytoliths are microscopic opaline (SiO_2_·nH_2_O) bodies found in many plant families and are especially prominent in the grass family [1]. Past research focused on the mechanism of their formation [2–4] and their use as proxies for past vegetation and climate [5–8]. Studies of the systematics of opaline phytolith morphologies have shown that many of these so-called morphotypes, especially in grasses, are in fact silicified replicas of plant cells or cell walls. Thus specific morphotypes reflect specific cells or tissues, and can be divided according to their origin within a plant (e.g., leaf vs. inflorescence phytoliths) and even serve as identifiers of plant species, genera and families [9]. This attribute in phytolith systematics is utilized to reconstruct past vegetation. In paleoecology, for example, phytolith morphotypes that are specific to C3 and C4 grasses are utilized to infer past climates [10]. In archaeology, phytoliths are used to detect concentrations of plant remains in archaeological sites [11], to determine the types of plants used by ancient humans [12], to detect (albeit with some controversy) the earliest appearance of domestic species such as maize and rice [13, 14], to recognize the types of plants utilized for fuel and bedding [15–17], and to identify spatial activities in archaeological sites [18]. With this abundance of research and its far-reaching implications for the past, one must understand the effect of partial dissolution on the interpretations that are derived from fossil phytolith morphotype assemblages.

Being an important source of silicon in the global terrestrial silicon cycle, a surge of interest in post-depositional alteration of phytoliths that involves partial dissolution is a hot research topic in recent years. Research in the last decade or so focuses on the role of phytoliths in the recycling of silicon by plants [19–21] which is tightly related to the mechanism of opal dissolution [22–26]. The latter studies highlight the fact that unlike previous assumptions, opaline phytoliths are relatively unstable in soils and sediments [27–32]. Yet, despite this realization little attention has been given by the paleoecological and archaeological research communities to the effect of partial dissolution on interpretations based on the morphological composition of fossil phytolith assemblages. Recently we pinpointed this issue by studying the effect of partial dissolution on the stability of phytoliths from domestic wheat plants [27]. Yet, the mechanism that explains which morphotypes would be stable or unstable following partial dissolution is currently unknown.

Several untested hypotheses have been raised to explain the differential stability of phytolith morphotypes. Bartoli and Wilding [33] identified Al in phytoliths and suggested that this impurity in the opal contributes to phytolith stability, a suggestion refuted by Fraysse *et al*. [23] and supported by Nguyen *et al*. [26]. The role of Al in stabilizing opal is thus not yet understood. Osterriech *et al*. [31] suggested that not only the presence of chemical impurities in phytolith opal, but also ‘maturity’ of a phytolith morphotype, may relate to phytolith preservation. They did not, however, supply criteria for assessing phytolith maturity. In addition, sediment pH, the specific silicified plant taxon and localities protected from weathering, were also suggested as parameters that influence phytolith preservation and affect interpretation of phytolith assemblages [1]. A most interesting observation was made by Wilding and Drees [34] who showed that biogenic opal produced in the leaves of deciduous trees is 10 to 15 times more soluble than opal produced by grasses. This observation led them to suggest that the larger surface to bulk ratio of tree leaf phytoliths make them more soluble than grass phytoliths. Fraysse, Pokrovsky, Schott and Meunier [23] studied the specific surface area in phytolith assemblages from 4 different plant species, showing that despite differences in specific surface area among the 4 plant species, the solubility of these phytolith assemblages was similar (ranging from 2.5 to 3.0 mM Si at pH 8 and 50°C). This indicates that specific surface area of a phytolith assemblage is not the main factor affecting phytolith solubility and relative stability. Additional research also highlighted the possibility that phytolith stability is related to the phytoliths’ surface to bulk ratio [31, 35], yet, no study to-date conducted direct measurements on individual phytoliths to test this hypothesis. To our knowledge, there is no currently available technique that enables direct measurement of specific surface area of single microscopic particles such as phytoliths, within an assemblage.

Given the vulnerability of phytolith assemblages to post-depositional partial dissolution and its critical effect on paleoecological and archaeological interpretation, it is surprising that the manner in which this process affects morphotype composition is poorly understood. Here we present a study that (a) explores the effect of partial dissolution on morphotype composition in 4 modern plant species, and (b) ties phytolith assemblage solubility and state of preservation to plant recycling of silicon and archaeological site formation processes. In addition, the data presented here may indicate that surface area to bulk ratio of individual phytolith morphotypes is an important factor governing differential phytolith stability. Our results establish that certain phytolith morphotypes which are significant for plant identification and thus for paleoecological and/or archaeological interpretation are subject to differential stability, and that preservation is best where deposited vegetal matter was rapidly detached from the terrestrial silicon cycle.

## Materials and Methods

### Phytolith extraction from fresh plants

Phytoliths were extracted from mature rice (*Oryza sativa L*. cv. Gleva) collected in a private field with the owner’s permission in Delta de l’Ebre, southern Catalonia (40°38’58.39”N 0°42’13.53”E). In addition, plant samples of reed (*Arundo donax*) were collected near Gvulot, southern Israel (31°13’42.57”N 34°30’25.94”E), and papyrus (*Cyperus papyrus*) and date palm (*Phoenix dactylifera*) were collected in Rehovot, central Israel (31°54’27.19” N 34°48’36.10”E) (S1 Table). The reed, papyrus and palm were collected in open areas where no need for permission is required. No endangered or protected species have been used in this study. Inflorescences were manually separated from leaves and stems. The plant parts were washed with distilled water and placed into a sonication bath for 15 minutes where surface mineral contamination was removed. This operation was repeated three times and the plant material dried at 50°C for 24 hours. Jenkins [29] demonstrated that dry ashing is the least damaging phytolith extraction method. Therefore about 50g of dry plant material was dry-ashed in a muffle furnace at 500°C for 4h. The ashes obtained were treated with 0.1N HCl to remove calcite. We used this highly diluted acid in order to avoid damage to the phytoliths, which were then centrifuged at 3000 rpm for 5 min and the supernatant was discarded. The operation was repeated three times with distilled water until all acid was removed from the samples. Centrifugation time was reduced as much as possible to avoid damage to delicate morphologies, although some breakage of conjoined phytoliths is expected [29]. The samples were then air-dried, weighed and their purity was assessed using Fourier Transform Infrared (FTIR) spectroscopy. This analysis enables detecting small amounts of clay and/or quartz that often attach onto plant surfaces as dust. FTIR analysis made it possible to ensure that these silicate minerals were not present in the opal samples studied. FTIR spectra were obtained by grinding a few mg of sample with a few tens of mg of KBr. Spectra were collected between 4000 and 400 cm^-1^ using a Nicolet 380 (Thermo Electron Corporation) and interpreted using reference libraries.

### Dissolution of phytoliths from fresh plants

The dissolution experiments were conducted in conditions similar to those reported in Cabanes *et al*. [27]. An aqueous solution buffered to pH 10 using 0.1 Trizma Base (Sigma) buffer and 0.1 M NaCl was prepared and the pH of the solution was adjusted using 1 M HCl. A weighed aliquot (around 5.5 mg) of the various pure phytolith samples were placed in separate 15 ml Falcon tubes, to which 6 ml of the buffer solution were added. The tubes were sealed with Parafilm and placed horizontally in an Innova 4230 shaker-incubator (New Brunswick Scientific, Edison, NJ, USA) set at 50°C and 70 rpm for 6 weeks. Each experiment was duplicated. Although in previous studies we stated that equilibrium may be reached after one week, here we studied phytolith assemblages from modern plants as well as from archaeological sites, thus as a precaution we carried out the experiments for 6 weeks until certain that all samples reached equilibrium.

Once a week the shaker-incubator was stopped, the tubes vortexed, centrifuged at 10,000 rpm for 10 min and 100 μl of solution was extracted from each tube and placed in 1.5 ml Eppendorf tubes for silicon content determination. 900 μl of distilled water were added to each tube, diluting the sample so that its concentration was within the measurement range of the instrument. Further dilution was conducted when necessary. The experiments were stopped at the end of the 6th week. The tubes were vortexed and centrifuged, the supernatant was transferred to a clean tube and stored for silicon content determination and the remaining phytoliths were washed three times with distilled water and centrifuged at 10,000 rpm for 10 min, dried in an oven at 50°C and stored for morphotype analysis.

Phytolith solubility was monitored by measuring the silicon concentration [Si] in the solution every week using the molybdate blue method [36] with an Ocean Optics USB–ISS–UV/VIS spectrophotometer. The accuracy of measurements is ±0.05 mM. Precision was determined from duplicate samples and is better than 0.2 mM.

### Dissolution of phytoliths from archaeological sites

Phytoliths were extracted from archaeological sediments collected at a ca. 3000 years old layer in the site of Tell es-Safi/Gath (Israel, 31°42'4.91"N 34°50'48.81"E) and a ca. 10,000 years old layer in the site of Aşıklı Höyük (Turkey, 38°20'57.13"N 34°13'47.10"E). All necessary permits were obtained for the described study, which complied with all relevant regulations. Samples from Tell es-Safi/Gath were collected under the supervision of A. M. Maeir, director of the excavation, under license no. G-68/2011 issued by the Israel Antiquities Authority. Samples from Aşıklı Höyük were collected under the supervision of Mihriban Ozbasaran, director of the excavation, under the permit no. 135328 issued by the Turkish Ministry of Culture and Tourism, General Directorate of Cultural Assets and Museums. Extraction followed a density-based separation procedure used for the rapid solubility test reported in *Cabanes et al*. [37]. Sample purity was assessed using FTIR spectroscopy. The purified phytolith assemblages were subject to partial dissolution in the same manner as the fresh phytolith assemblages, followed by measurement of [Si] using a UV-VIS spectrophotometer as described above.

### Analysis of phytolith morphotypes from fresh plants

The calculation of phytolith concentration in 1 mg of sample as well as phytolith morphotype identification was carried out before and after dissolution using a petrographic microscope (Olympus BX41) at 200× and 400×. Between 0.5 to 1 mg of phytolith residue at the beginning and end of each experiment was mounted on microscope slides using Entellan New (Merck). Two slides were prepared from the initial residue (i.e., before dissolution) and two slides from the final residue (i.e., after dissolution). Each slide was initially studied at 200x magnification and phytoliths were counted in 16 random fields. Phytolith concentrations were calculated following Albert and Weiner [38]. At least 250 individual phytoliths were classified to morphotypes in each slide. Phytoliths in anatomical connection (multicellular structures) were identified and quantified individually and added to the general individual count. Identification of phytolith morphotypes was carried out using the standard literature [1, 9, 39, 40]. The plants used in the experiments have been selected because of their ecological and archaeological importance. Some of these plants have distinctive phytolith morphotypes that can be identified to genera or species level (i.e., double husk points in rice, spheroid echinates in palms, or hat-shape phytoliths in sedges), but they also include morphotypes which are commonly used to reconstruct past vegetation. Therefore, we focus our presentation of the results on the following morphotypes: bulliform cells, double peaked rice husk cells, hat-shaped sedge phytoliths, elongate parallelepipedal phytoliths, bilobate and rondel short cells, and echinate spheroid palm phytoliths. Other phytoliths, such as weathered morphotypes, are considered in order to better understand the changes produced by partial dissolution.

### Geometric surface area to bulk ratios

Several researchers pointed out the possible importance of surface area to bulk ratios for individual phytolith stability [31, 33, 35]. In the absence (to our knowledge) of an analytical method that can accurately determine the chemically active surface area of individual phytoliths within an assemblage, we provide here a crude calculation of the microscopic surface area to bulk ratio of individual phytoliths. Because gross differences in surface to bulk ratios are primarily dependent on shape and size, we selected several morphotypes (S1 Table) whose importance for ecological and archaeological research is known and conducted direct measurements of a representative sample of individual phytoliths. The results were compared to the differences observed in phytolith morphotype absolute concentrations and relative representation after dissolution.

Phytoliths were immersed in liquid Entellan on microscope slides which allowed their rotation using gentle force via a pencil tip. Phytoliths were rotated to positions that were perpendicular to the field of view so that parameters such as length, width and height can best be captured using an Olympus DP71 equipped with a camera and measured with *cell^D* software which allows high precision measurements to be conducted. The precision of measurements was better than 1μm, assessed as the standard deviation from an average that was produced by repeating the measurements 10 times on the same phytolith (done in 10 phytoliths). At least 10 different individuals from each morphotype were measured, followed by calculation of their surface area (SA) and volume (V). Because phytolith morphotypes are not formal three-dimensional geometric shapes, we calculated the SA and V of the closest possible three-dimensional geometric shapes (see S1 Table). These calculations provide the best approximation of SA and V of the studied morphotypes and allow calculation of the SA/V ratio. Due to these approximation calculations, the SA/V in decorated morphotypes (e.g., wavy long cells and rice double-peaked husk) reflects minimum values. We note that relatively large variability exists in these calculations per morphotype (i.e., the 1σ standard deviation from the average) which primarily reflects the natural variability in phytolith sizes in an assemblage (see S1A Fig – double peak husk from rice inflorescence—and S1P Fig – hat-shape from sedge inflorescence, for an example of natural variability of sizes among the same phytolith morphotype in the same assemblage1). Note that these calculations consider geometry only, henceforth regarded as ‘geometric surface to bulk ratios’, while if future analyses will allow direct measurements of the chemically active surface area of individual phytoliths we expect the values to be much higher. The correlation we observe (see below) between morphotype stability and SA/V values indicates that our approximations probably reflect a real causation.

## Results

### Solubility of phytolith assemblages

**Fig 1:**
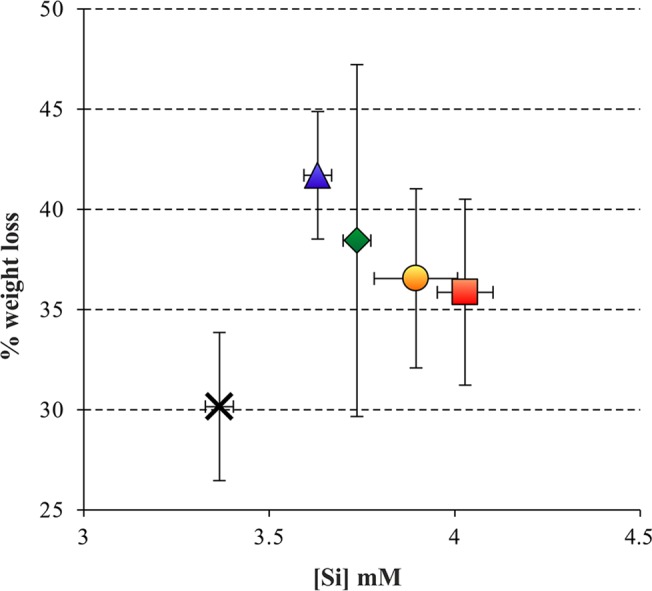
The solubility of phytolith assemblages extracted by dry ashing from modern fresh plant samples. Solubility was determined at pH 10 after the solutions reached saturation with respect to Si. The percentage of weight loss shows that the experimental conditions resulted in partial dissolution of the phytolith assemblages. Error bars indicate 1σ standard deviation between duplicates. Cross: sedge inflorescence; triangle: reed leaf; diamond: rice inflorescence; circle: palm leaf; square: rice leaf.

**Fig 2:**
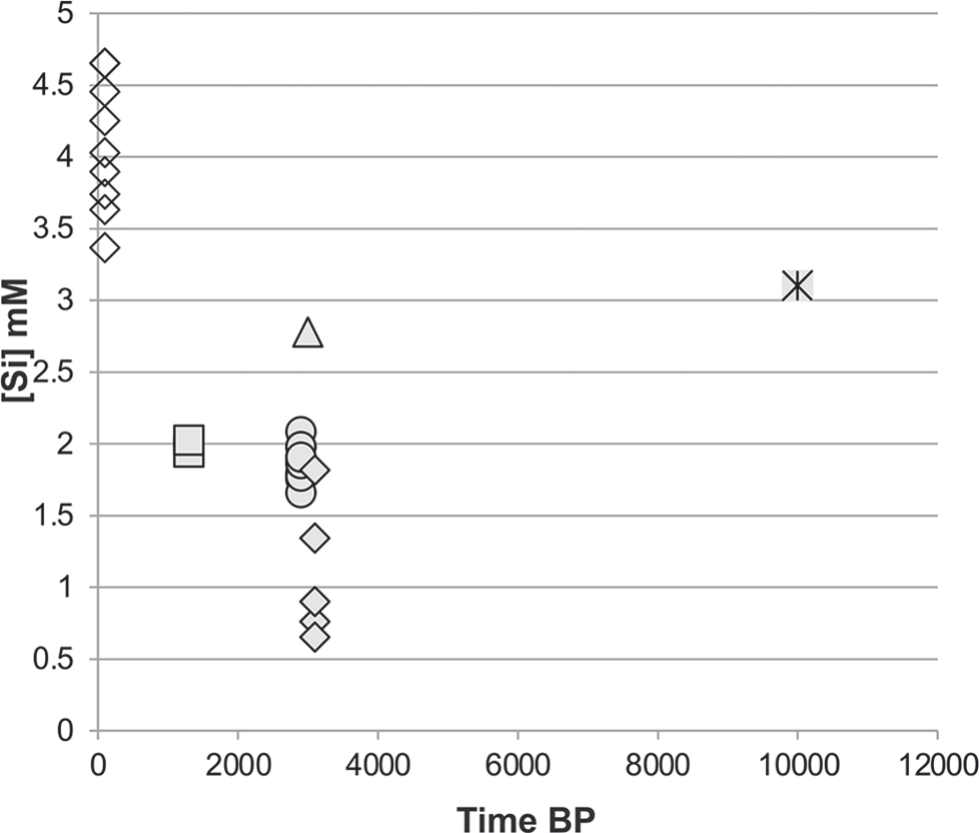
Comparison of solubility at pH 10 among modern and ancient phytolith assemblages in relation to antiquity. Open diamonds: modern plants (this study and wheat from Cabanes *et al*.[27]). Squares: 7^th^ century AD assemblages from the shallowly buried site of Wadi el-Mustayer, Negev desert, Israel [40]. Circles: 9^th^ century BCE assemblages from the deeply buried site of Tell es-Safi/Gath, southern Shephela, Israel. Triangle: 10^th^ century BCE assemblage from the deeply buried site of Tel Dor, northern coast, Israel [27]. Full diamonds: 11^th^ century BCE assemblages from the shallowly buried site of Izbet Sartah, western Samarian hills, Israel [37]. Star: 9^th^-mid 8^th^ millennia BCE assemblages from the deeply buried site of Aşıklı Höyük, Anatolia, Turkey. Note that high solubility at pH 10 indicates better preservation as solubility is closer to that of modern plant assemblages. The lowest values, indicating poor preservation, were obtained from shallowly buried sites from humid Mediterranean environments. The highest values, indicating good preservation, were obtained from deeply buried sites in various environmental settings with no relation to antiquity. Exceptional good preservation is noticed in shallowly buried sites in arid environments.

The solubility of whole phytolith assemblages is important for evaluating whether or not basal differences exist among modern and fossil contexts. Such measurements are available but were conducted using several extraction and dissolution methods [22–25], making it difficult to compare results among studies. We report solubility measurements conducted at pH 10 using an assay that also yielded past results [27, 41], which enables comparison between and within modern and fossil assemblages. The solubility of the 5 modern plant phytolith assemblages reported here (S1 Table) range between 3.3 and 4.1 mM Si, in accordance with previously published data [27] (Fig 1). The solubility of fossil phytoliths extracted from sediments from two archaeological sites range from 1.6 to 2.1 mM Si in Tell es-Safi/Gath (Israel; n = 11) and 3.2 in Aşıklı Höyük (Turkey, n = 2). These values together with solubilities measured using similar methodology in various other archaeological sites in the Near East are presented in relation to the antiquity of these samples (Fig 2), showing that phytolith solubility in modern plants clusters at higher values than the solubility values of fossil phytolith assemblages. We interpret this to indicate that while modern phytolith assemblages include a large amount of unstable phytoliths, the fossil assemblages release less silicon upon alkaline conditions because they are composed of rather stable phytoliths. This observation indicates that partial dissolution will be evident through morphotype analysis [27, 37].

### Morphotype changes after partial dissolution

**Fig 3:**
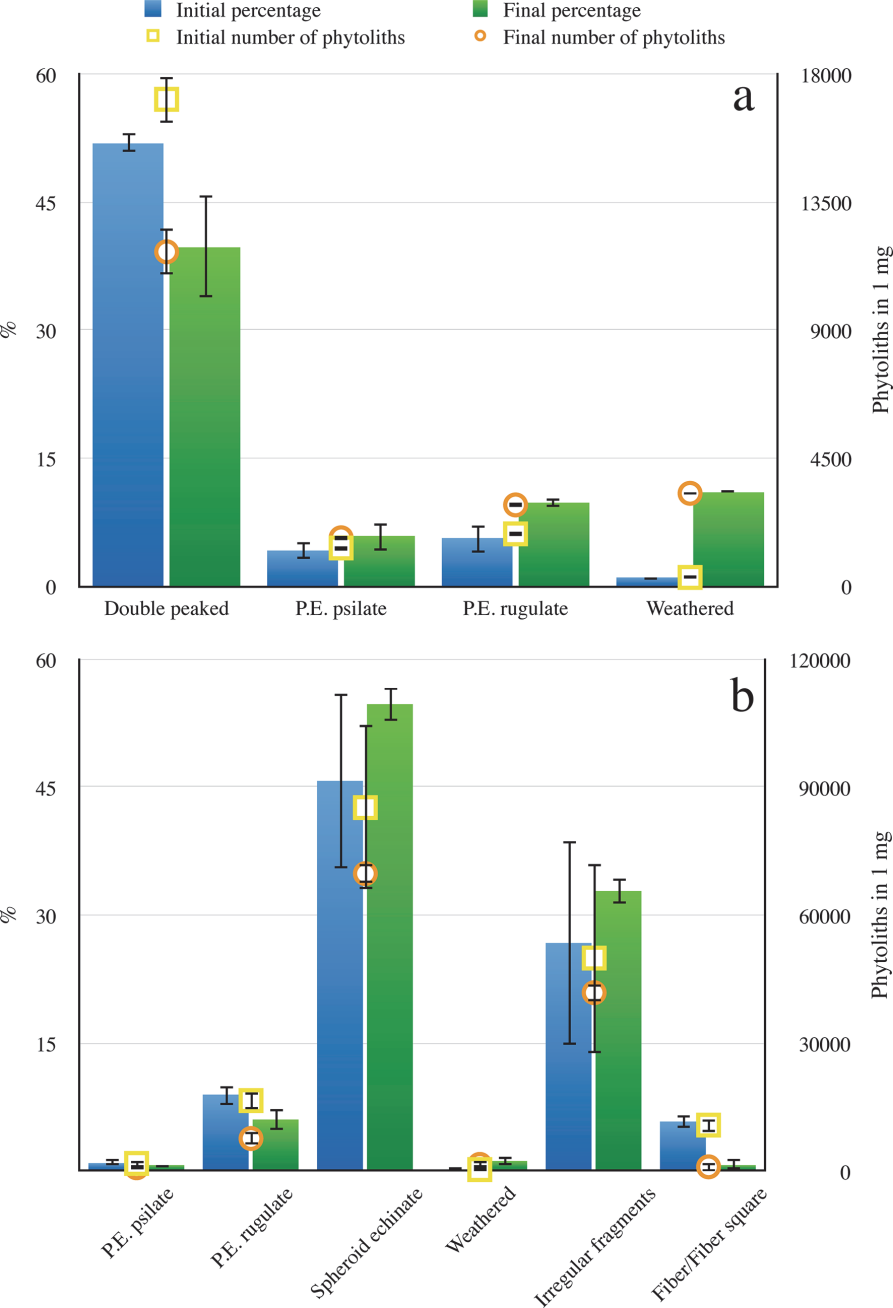
Changes in absolute concentrations and relative proportions of the most abundant phytolith morphotypes. Changes in the absolute contenctrations (left axis) and relative proportions (right axis) in two examples of plant phytoliths studied: most abundant morphotypes in two examples from the phytolith assemblages studied: **a,** Rice inflorescence. **b,** Date palm leaf. Light grey bars: initial phytolith concentration (number in 1mg opal powder); dark grey bars: final phytolith concentration (number in 1mg opal powder); circles: initial relative abundance (%); squares: final relative abundance (%). Error bars indicate 1σ standard deviation between duplicates. Note that the absolute concentration of phytoliths, as well as the relative abundance of morphotypes may either decrease, increase or stay unchanged, with all combinations possible. This attests for differential stability of morphotypes.

So far only one study, conducted on domestic wheat phytolith assemblages, quantified the fate of phytolith morphotypes after partial dissolution [27], showing that the relative abundance of certain morphotypes either increases, decreases or remains unchanged. The general patterns observed in selected phytolith morphotypes in the 5 plant assemblages reported here relate to the absolute phytolith concentrations before and after partial dissolution, and to changes in the relative abundance (percentage) of morphotypes. In general, absolute quantities decrease in most studied morphotypes after partial dissolution (Fig 3; see also S2–S5 Figs). This decrease may be accompanied by either decreased (e.g., double-peaked husk phytoliths in rice inflorescence, Fig 3A), or unchanged relative abundance (e.g., bilobate short cells in rice leaves, S2 Fig). At the same time, an increase in absolute concentrations is observed in weathered morphotypes, opaline fragments which can no longer be assigned to specific morphotypes, and in morphotypes with rugulate (rugose) surfaces (the latter due to loss of the original surface decoration [27] and thus an indication for partial dissolution; e.g., P.E. rugulate and weathered morphotypes in Fig 3A). Certain other morphotypes may remain unchanged in both parameters (e.g., spheroid/globular echinate, Fig 3B). This variability in the effect of partial dissolution on absolute concentrations and relative abundance of phytoliths indicates that although all are composed of the same mineral and have a similar range of solubility, the stability of the various morphotypes differs.

### Geometric surface area to bulk ratio in selected morphotypes

**Fig 4:**
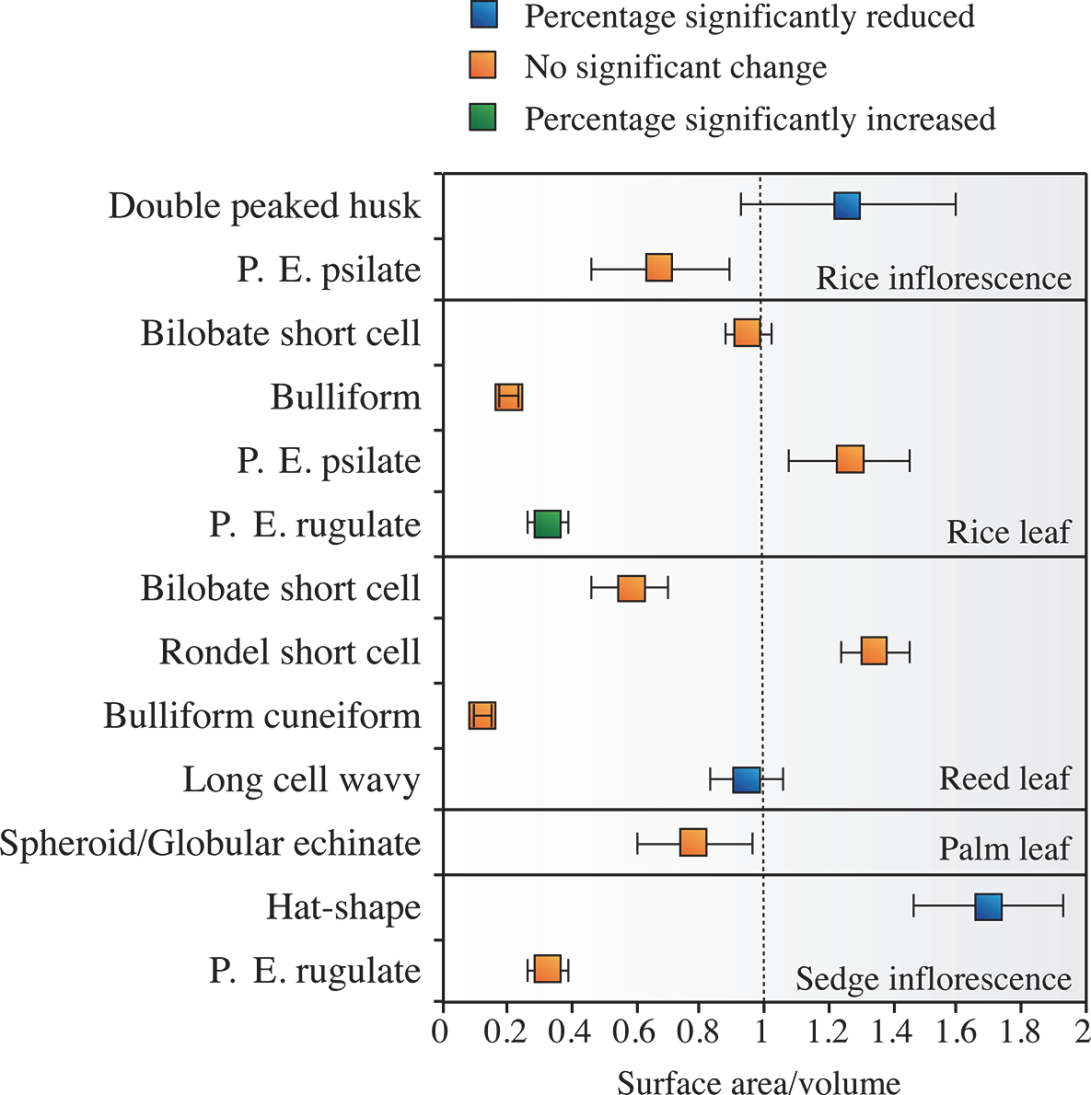
Calculated surface area to bulk (SA/V) ratios for selected morphotypes in the various modern plant assemblages. Error bars indicate 1σ standard deviation among several measured individual phytoliths of the same morphotype (S1 Table). The relatively large variation reflects the natural variability in phytolith sizes. Values above 1 indicate relatively unstable morphotypes while values below 1 indicate relatively stable morphotypes. Note that the same morphotype (e.g., psilate parallelpipedal elongated, here denoted as P.E. psilate) may have very different SA/V ratio in different plant parts (or in different plant species, e.g., bilobate short cells from rice leaves vs. reed leaves), depending on their overall size—the larger the morphotype the lower its SA/V value and the higher its stability. This observation indicates that bulkiness, as affected by size, are important factors determining the stability of phytolith morphotypes.

The results of geometric surface to bulk ratios are shown in Fig 4 and S1 and S2 Tables. Note that the SA/V values do not reflect accurate measurements of the chemically active surface area, or the volume, yet, when compared to the differences observed in absolute concentrations and relative proportions of the morphotypes analyzed, certain correlations seem to be present: Morphotypes with a geometric surface to bulk ratio below 1 (e.g., bulliform cells from rice and reed leaves,) are also those whose percentages and absolute concentration did not decrease significantly following partial dissolution (compare Fig 4 with Fig 3 and S2–S5 Figs). These are the largest and more voluminous morphotypes. On the other hand, morphotypes with a surface to bulk ratio higher than 1 (e.g., double-peaked husk phytoliths from rice and hat-shaped phytoliths from sedges) are those whose percentages and absolute concentrations decreased significantly following partial dissolution (Fig 4). These are rather small-sized morphotypes. Yet, spherical forms, such as those of globular echinates from palm phytoliths, despite their small size have a geometric surface to bulk ratio close to 1, and indeed these morphotypes remain unchanged following partial dissolution (Fig 4). In bilobate short cells, we note that while these are rather stable morphotypes based on unchanged relative abundance values, their geometric surface to bulk ratio may be quite different depending on size, where it is close to 1 in the smaller-sized bilobates from rice leaves and around 0.5 in the larger-sized bilobates from reed leaves (Fig 4 and S2 Table). An exception to the pattern thus revealed is noted for rondel short cells in reed leaves which have a geometric surface to bulk ratio higher than 1 and their size is small rendering them susceptible to dissolution, yet their relative abundance did not change significantly following partial dissolution (Fig 4). A possible explanation is their presence within multicellular structures supplying physical protection from dissolution (S1G and S1H Fig).

Clearly, the patterns observed indicate that more than one parameter affects the differential stability of phytolith morphotypes. The SA/V values should not be taken as accurate measurements, rather, as crude markers for bulky vs. delicate morphotypes. Still, the relationship between shape and size as revealed through the geometric ratio of surface area to bulk in individual phytoliths seen here explains most of our observations in the experimental phytolith dissolution, and can thus be regarded as the major detriment parameter on the differential stability of individual phytoliths. This underlying mechanism produces distorted morphotype compositions in partially dissolved phytolith assemblages. Below we discuss the implications of individual morphotype differential stability for paleoecologcial and archaeological interpretation that is based on moprhotype representation in whole phytolith assemblages.

## Discussion

Our data suggests that the microscopic (geometric), rather than nanoscopic (specific) surface area of phytolith morphotypes is an important factor that affects, among others, the likelihood of phytolith preservation. In this respect, our results accord with those reported by Wilding and Drees [34]. Others suggested that Al impurities, phytolith maturity and specific surface area are important factors to phytolith preservation, but results seem contradictory among previously published studies [23, 26, 33]. The mechanism that determines phytolith stability throughout archaeological and geological time scales should be studied further.

The comparable whole assemblage solubility data (Fig 2) indicates that the lower the solubility of a given phytolith assemblage, the more it is composed of resistant phytoliths, i.e., it is less well preserved. This in turn, supplies us with a new tool for determining the state of preservation of fossil phytolith assemblages, which is extremely important for paleoecological and archaeological reconstructions in light of the data presented here. We advocate the use of a rapid solubility assay [37] that is based on the methodology by which the data in Fig 2 was obtained. Using the scheme presented in Fig 2 coupled with data from publications cited therein, we suggest that solubility values below 2mM [Si] indicate poor preservation (i.e., highly distorted morphotype composition), values between 2–3 mM [Si] indicate good preservation (i.e., somewhat distorted assemblages), and values above 3mM [Si] indicate excellent preservation (i.e., slightly or un-distorted assemblages).

The distortions expected if poorly preserved phytolith assemblages are used for paleoecological reconstruction include: (1) Inaccurate assessment of water availability/stress to plants and thus climate, often based on percentages of bulliform morphotypes [42] which will be over-represented following partial dissolution. (2) Past vegetation in relation to climate change is often estimated based on the ratio of grass short cells [5, 6, 38, 43]. The results presented here show that bilobate short cell are fairly stable and that rondels locked within multicellular structures may be well preserved even though their geometric surface/bulk ratio is higher than 1. The stability of short cells is complex to the degree that it is uncertain, at this point in research, what governs their representation in fossil assemblages. More research is needed to elucidate this. (3) Reconstruction of the Plio-Pleistocene landscapes in East Africa [28, 44, 45] from poorly-preserved assemblages may be distorted due to the instability of hat-shaped sedge phytoliths on the one hand, and relative stability of spheroid/globular echinate phytoliths from palm leaves on the other.

In archaeology, phytolith morphotypes are often used to infer a wide variety of past human-plant interactions. The ratios of dicotyledonous and monocotylendonous phytoliths in archaeological assemblages, often used to reconstruct selection of fuel materials by ancient humans [46], will be distorted following partial dissolution [34]. Partial dissolution of phytolith assemblages may also affect the identification of agricultural activities in archaeological sites. The ratio of inflorescence to leaf-stem phytoliths, used to identify location of storage or crop-processing activities [11, 47], may be distorted in favor of leaf-stem to inflorescence phytoliths [27], which would further affect identification of activity areas. In rice, the double-peaked husk phytolith used to differentiate rice to the species level [48], is a rather delicate morphotype, expected to be under-represented in a poorly-preserved phytolith assemblage. On the other hand, the rice cuneiform bulliform cells, whose morphometrics are used to distinguish domestic from wild rice [49] are rather stable and can safely be used in this respect. In a previous study [27] we have shown that the dendrites in dendritic long cell phytoliths from domestic wheat are highly soluble so that they may be mistakenly identified as echinate long cell phytoliths, after partial dissolution. Their importance lies in the fact that the identification of domestic wheat and barley is sometimes based on the relative abundance of dendritic long cell phytoliths [11] and/or on their morphometric characteristics [50]; values that might be severely distorted due to partial dissolution. Lastly, the abundance and size of multicellular structures [51] used to reconstruct irrigation of ancient crop fields are prone both to mechanical breakage [29] and dissolution. Clearly, the effect of post-depositional partial dissolution must be considered in future studies seeking to accurately reconstruct the complex inter-relationships between humans and their environments.

The comparative scheme for assessment of the state of preservation of whole phytolith assemblages (Fig 2) is clearly useful for pre-screening of phytolith assemblages for vegetation reconstruction purposes. Moreover, it opens a window into understanding the dependency of the state of preservation of phytolith assemblages on time and environmental factors. In soils, solubility of phytolith assemblages decreases with depth [19, 20, 52] indicating that the deeply buried phytoliths are poorly preserved and that preservation is time dependent. In archaeological sediments the situation seems to be more complex (Fig 2). (a) The best preserved phytolith assemblages originate from an archaeological layer dated to ca. 10,000 years ago (at Aşıklı Höyük). Another well-preserved sample dates to ca. 3000 years ago (at Tel Dor), while the youngest sample is moderately well-preserved (at Wadi el-Mustayer). (b) Phytolith assemblages dating to about the same time, from different archaeological sites, show a wide range of variation in solubility. These two observations indicate that phytolith preservation in archaeological sites is not time dependent. Moreover, (c) Phytolith assemblages from deeply buried archaeological layers seem to be better preserved than assemblages obtained from shallowly buried sites, dating to the same period (compare the deeply buried Tel Dor and Tell es-safi/Gath assemblages with the shallowly buried Izbet Sartah assemblage). (d) Phytolith assemblages from shallowly buried archaeological layers of different periods are better preserved in an arid environment (compare the arid Wadi el-Mustayer assemblage with that of the Mediterranean Izbet Sartah assemblage, both shallowly buried sites). The latter two observations indicate that the state of preservation of fossil phytolith assemblages depends on the depth of burial and water availability at the burial environment. These factors are associated with the terrestrial silicon cycle in which phytoliths are recycled by plant roots immediately upon their deposition in the soil A horizon [19, 20]. Cabanes *et al*. [37] noted that if plant material was rapidly deeply buried, as may occur in certain archaeological sites, the detachment of phytoliths from soil formation processes (via detachment from plant root activity) enhances their chances of preservation. This highlights the importance of rapid burial, a factor known to be responsible for good preservation of many other types of fossils and archaeological remains [53]. Therefore where soil development is rather slow and under constant biomass activity, the preservation of phytolith assemblages worsens with depth. In the shallow portions of soil profiles in humid environments phytolith recycling is constantly active resulting in poor state of phytolith preservation. In arid environments where biomass is low and soils develop very slowly, if at all, phytoliths preserve even in the shallow portions of the soil, as well as in shallowly buried open-air archaeological sites. We note that in cave or rockshelter environments, where plant growth is minimal or absent, phytoliths are detached from the terrestrial silicon cycle. Yet, in such environments pH can be predominantly alkaline (especially in highly active karstic environments) which raises the chances of phytolith dissolution via a purely chemical mechanism [15, 54].

## Conclusions

We have shown here evidence for differential stability of various phytolith morphotypes from four ecologically and archaeologically important plant species. We suggest that one of the factors that determines this differential stability relates to the geometric surface to bulk ratio of individual phytoliths which is tightly related to morphotype shape and size. This new understanding helps to evaluate the reliability of certain morphotypes for ecological and archaeological reconstruction, cautioning that interpretations obtained from poorly preserved phytolith assemblages present distorted botanic scenarios. We stress that phytolith state of preservation should be estimated prior to reconstruction studies, preferably using a universal solubility test (as suggested above). Furthermore, we show that a biogeochemical mechanism, not dependent on length of time but primarily on rate of burial and the availability of water, determines the state of preservation of phytolith assemblages in soils and sediments. Future research should target other plant species that are important for paleoecological and archaeological research, such as maize, banana or squash, and seek to develop new understandings on the mechanism that leads to differential stability in phytolith assemblages. In addition, analytical developments will be necessary to accurately measure the surface to bulk ratio of individual phytolith morphotypes. Such studies will have an especially important impact on archaeological interpretation and human-plant interactions throughout the human career, where phytolith analysis is increasingly used and valued.

## Supporting Information

S1 Fig Images of the main morphotypes referred to throughout the text. **a**, Double-peaked husk phytolith from rice inflorescence before partial dissolution. **b**, Double-peaked husk phytolith from rice inflorescence with an alteration at the base of the morphotype after partial dissolution. **c,** Cuneiform bulliform cell from reed leaves before partial dissolution. **d,** Altered cuneiform bulliform cell from reed leaves after partial dissolution. **e,** Parallelepipedal elongate rugulate phytolith from rice inflorescence before partial dissolution. **f**, Altered parallelepipedal elongate from rice inflorescence after partial dissolution. 1 and 2: weathering at the margins of the morphotype, 3: changes on the surface of the morphotype from psilate to rugulate. **g**, Morphotypes from reed leaves before partial dissolution. 1: Bilobate short cells. Note their larger size relative to those in rice leaves. 2: Rondel short cells embedded within a multicellular structure that also includes wavy long cells, 3. **h**, multicellular structure formed by heavily altered long cells and bilobate short cells after partial dissolution. Note how short cells in this case seem to be more stable than long cells. **i,** Bilobate short cells from rice leaves before partial dissolution. **j,** Bilobate short cell from rice leaves with distal alteration after partial dissolution. **k,** Parallelepipedal elongate psilate phytolith from rice leaves before partial dissolution. **l,** parallelepipedal elongate with altered surface from rice leaves after partial dissolution. **m,** Cuneiform bulliform cell from rice leaves before partial dissolution. **n,** Weathered cuneiform bulliform from rice leaves after partial dissolution. **o,** Multicellular structure formed by three spheroid/globular echinate phytoliths from date-palm leaves before partial dissolution. **p,** Hat-shaped phytoliths from sedge inflorescence before partial dissolution. 1: Side view. 2: Top view. 3. Hat-shaped phytoliths in anatomical connection.
(JPG)

S2 Fig Changes in concentrations and percentages of phytolith morphotypes in rice leaves. P.E. denotes parallelepipedal elongate phytoliths. Note increase in the percentage of P.E. rugulate and decrease in papillateae and verrucate/corniculate long cell phytoliths, following partial dissolution.
(TIF)

S3 Fig Changes in concentrations and percentages of phytolith morphotypes in reed leaf. P.E. denotes parallelepipedal elongate phytoliths. Note decrease in the wavy long cells, mesophyll phytoliths, rondel and bilobate short cells and increase in weathered phytoliths, following partial dissolution.
(TIF)

S4 Fig Changes in concentrations and percentages of phytolith morphotypes in rice leaves and sedge inflorescence. Note the significant decrease in both percentage and absolute concentrations in the distinctive hat-shaped phytoliths, as well as in P.E. psilate and the sinuous long cell morphotypes, while mesophyll and P.E. rugulate morphotypes decrease in absolute concentration but their percentages remain unchanged, following partial dissolution.
(TIF)

S5 Fig Changes in the multicellular structures of modern rice, reed, palm and sedge. Light grey vertical bars: percentage of phytolith in anatomical connection before partial dissolution; dark gray vertical bars: percentage of phytoliths in anatomical connection after partial dissolution; circles: average number of phytoliths forming a conjoined multicellular structure before partial dissolution; squares: average number of phytoliths forming a conjoined multicellular structure after partial dissolution. Note the general reduction in the percentage of anatomically connected phytoliths, but the unchanged average amount of connected phytoliths per structure, following partial dissolution.
(TIF)

S1 Table Approximate ratios of geometric surface area to volume in selected morphotypes. *Footnote*: P.E. denotes elongated parallelepipedal phytoliths.
(DOCX)

S2 Table Raw measurements used for the calculation of the geometric surface to volume ratio. Measurements are expressed in μm, while surface area is expressed in μm^2^ and volume in μm^3^. P.E. denotes elongated parallelepipedal phytoliths.
(DOCX)
