# Supplementary figures and images for: Understanding Fossil Phytolith Preservation: The Role of Partial Dissolution in Paleoecology and Archaeology
Source: PLoS One. 2015 May 20;10(5):e0125532. doi: 10.1371/journal.pone.0125532 (PMC4439089; doi:10.1371/journal.pone.0125532)

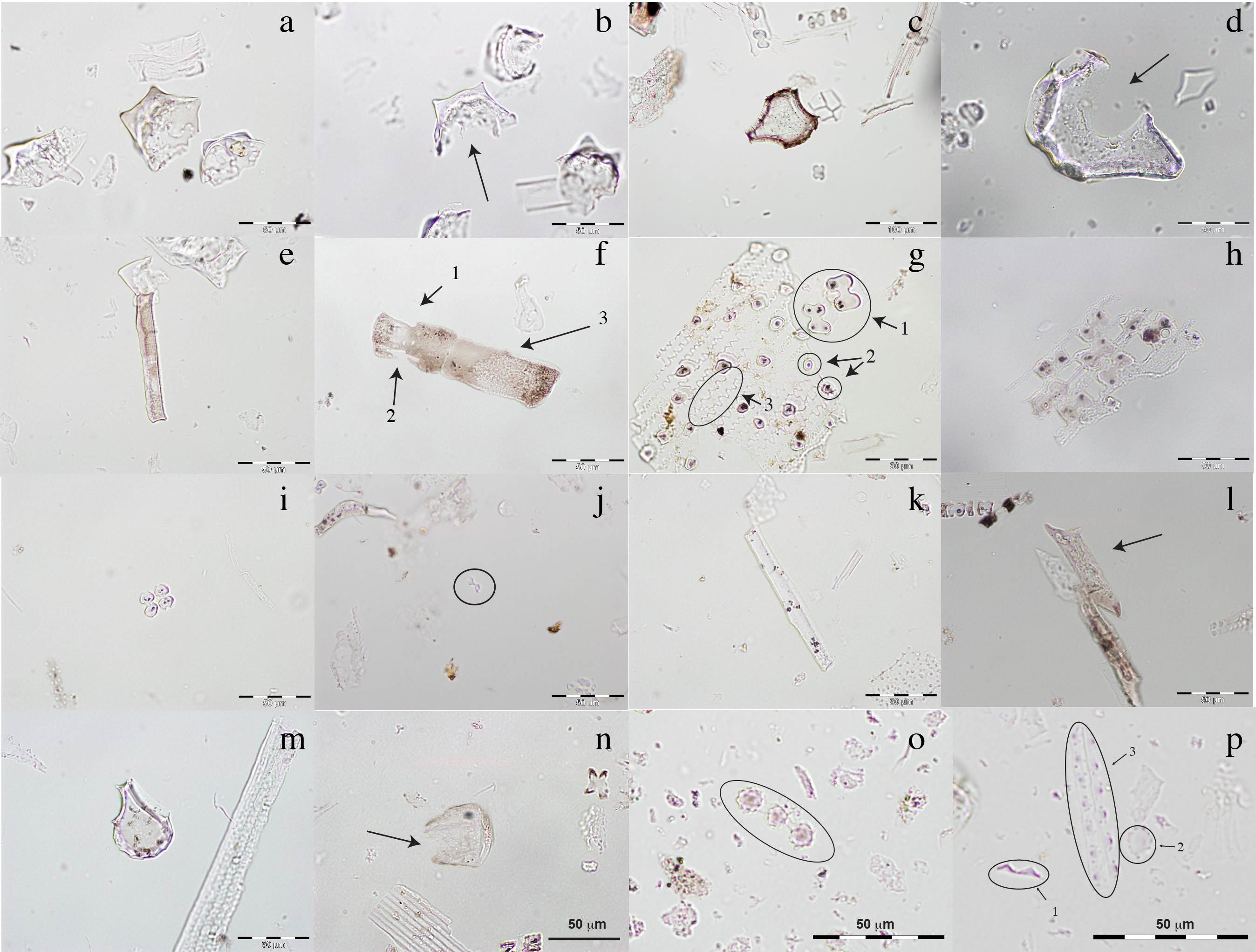

Supplement: S1 Fig — a, Double-peaked husk phytolith from rice inflorescence before partial dissolution. b, Double-peaked husk phytolith from rice inflorescence with an alteration at the base of the morphotype after partial dissolution. c, Cuneiform bulliform cell from reed leaves before partial dissolution. d, Altered cuneiform bulliform cell from reed leaves after partial dissolution. e, Parallelepipedal elongate rugulate phytolith from rice inflorescence before partial dissolution. f, Altered parallelepipedal elongate from rice inflorescence after partial dissolution. 1 and 2: weathering at the margins of the morphotype, 3: changes on the surface of the morphotype from psilate to rugulate. g, Morphotypes from reed leaves before partial dissolution. 1: Bilobate short cells. Note their larger size relative to those in rice leaves. 2: Rondel short cells embedded within a multicellular structure that also includes wavy long cells, 3. h, multicellular structure formed by heavily altered long cells and bilobate short cells after partial dissolution. Note how short cells in this case seem to be more stable than long cells. i, Bilobate short cells from rice leaves before partial dissolution. j, Bilobate short cell from rice leaves with distal alteration after partial dissolution. k, Parallelepipedal elongate psilate phytolith from rice leaves before partial dissolution. l, parallelepipedal elongate with altered surface from rice leaves after partial dissolution. m, Cuneiform bulliform cell from rice leaves before partial dissolution. n, Weathered cuneiform bulliform from rice leaves after partial dissolution. o, Multicellular structure formed by three spheroid/globular echinate phytoliths from date-palm leaves before partial dissolution. p, Hat-shaped phytoliths from sedge inflorescence before partial dissolution. 1: Side view. 2: Top view. 3. Hat-shaped phytoliths in anatomical connection. (JPG) [file pone.0125532.s001.jpg]

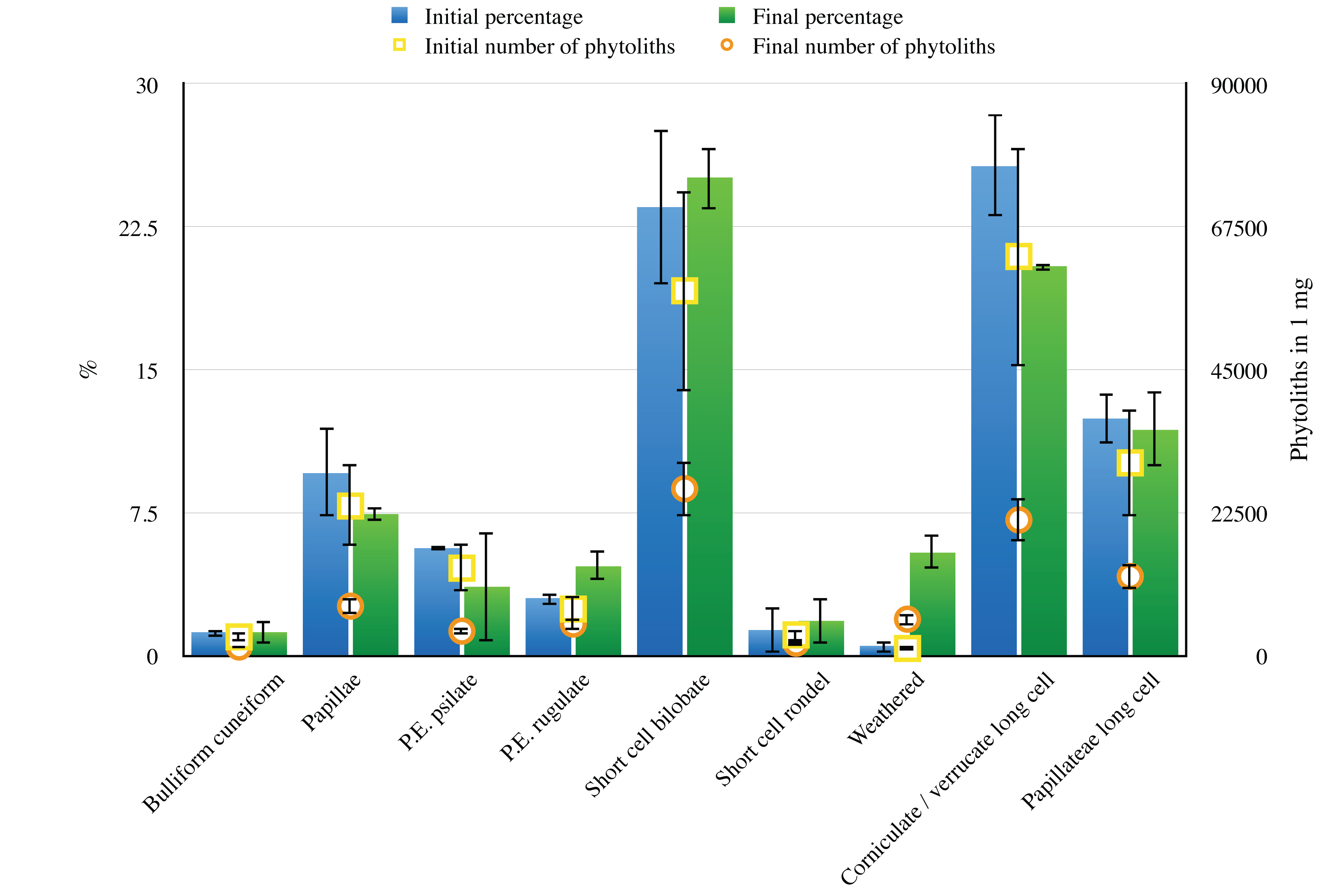

Supplement: S2 Fig — P.E. denotes parallelepipedal elongate phytoliths. Note increase in the percentage of P.E. rugulate and decrease in papillateae and verrucate/corniculate long cell phytoliths, following partial dissolution. (TIF) [file pone.0125532.s002.tif]

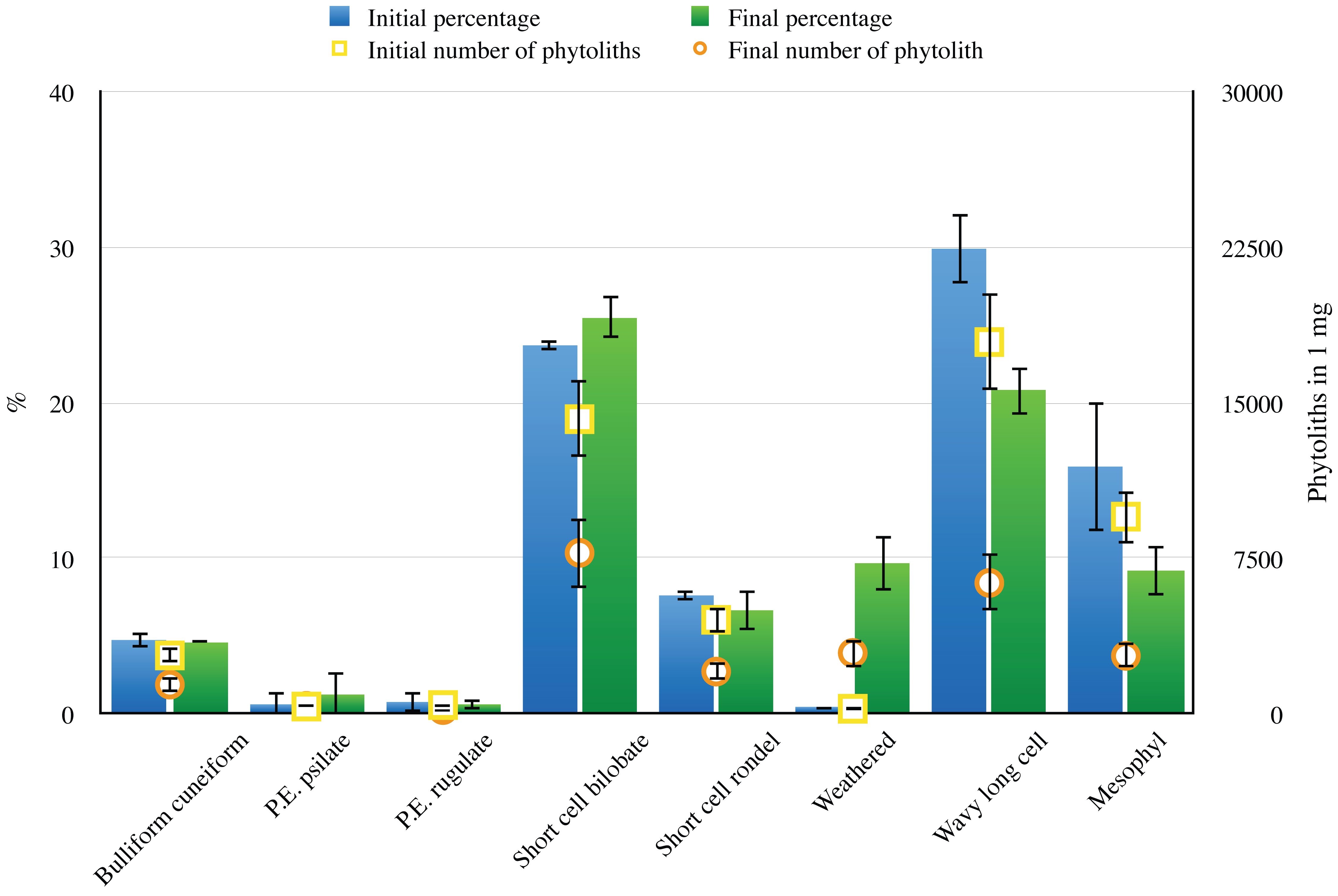

Supplement: S3 Fig — P.E. denotes parallelepipedal elongate phytoliths. Note decrease in the wavy long cells, mesophyll phytoliths, rondel and bilobate short cells and increase in weathered phytoliths, following partial dissolution. (TIF) [file pone.0125532.s003.tif]

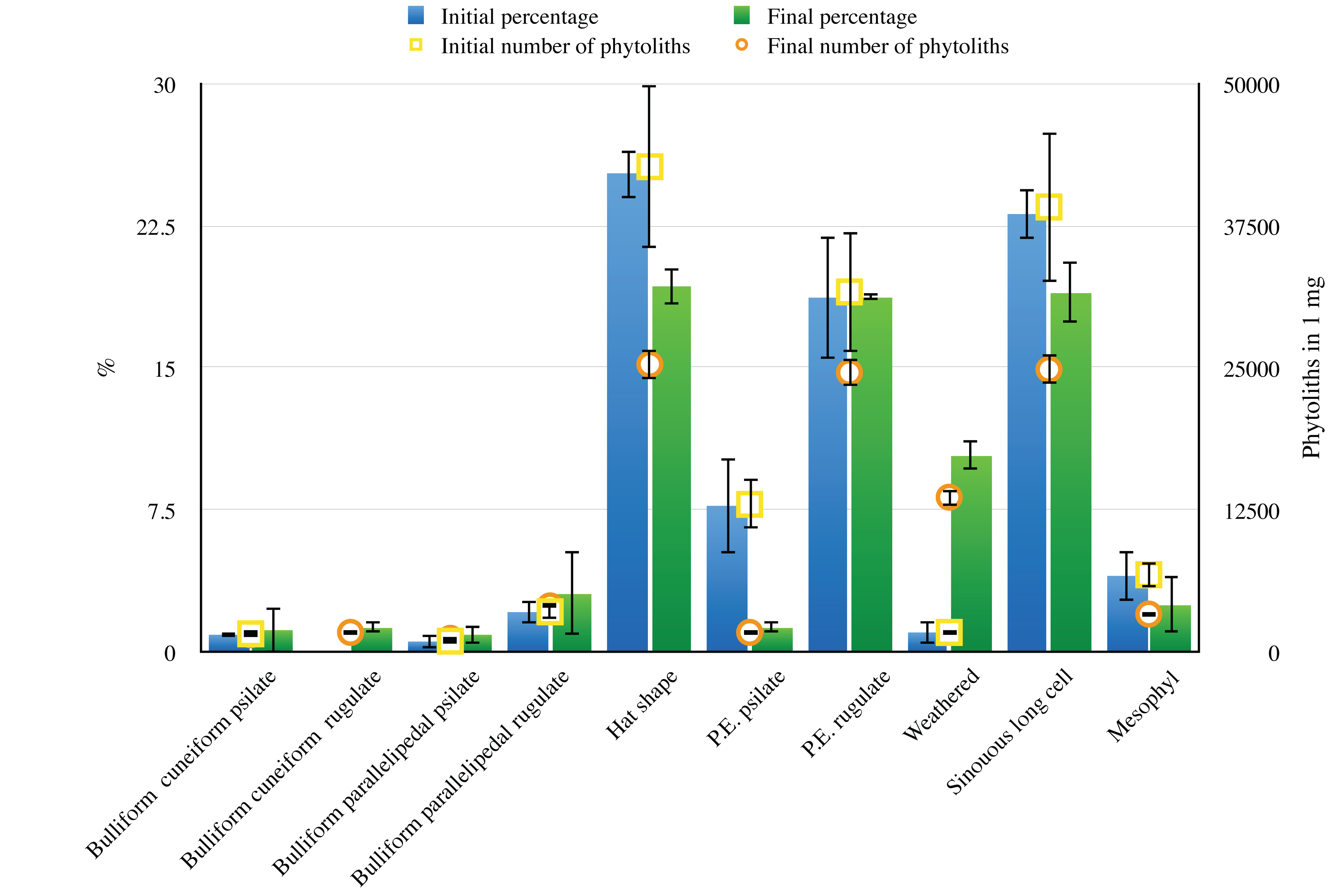

Supplement: S4 Fig — Note the significant decrease in both percentage and absolute concentrations in the distinctive hat-shaped phytoliths, as well as in P.E. psilate and the sinuous long cell morphotypes, while mesophyll and P.E. rugulate morphotypes decrease in absolute concentration but their percentages remain unchanged, following partial dissolution. (TIF) [file pone.0125532.s004.tif]

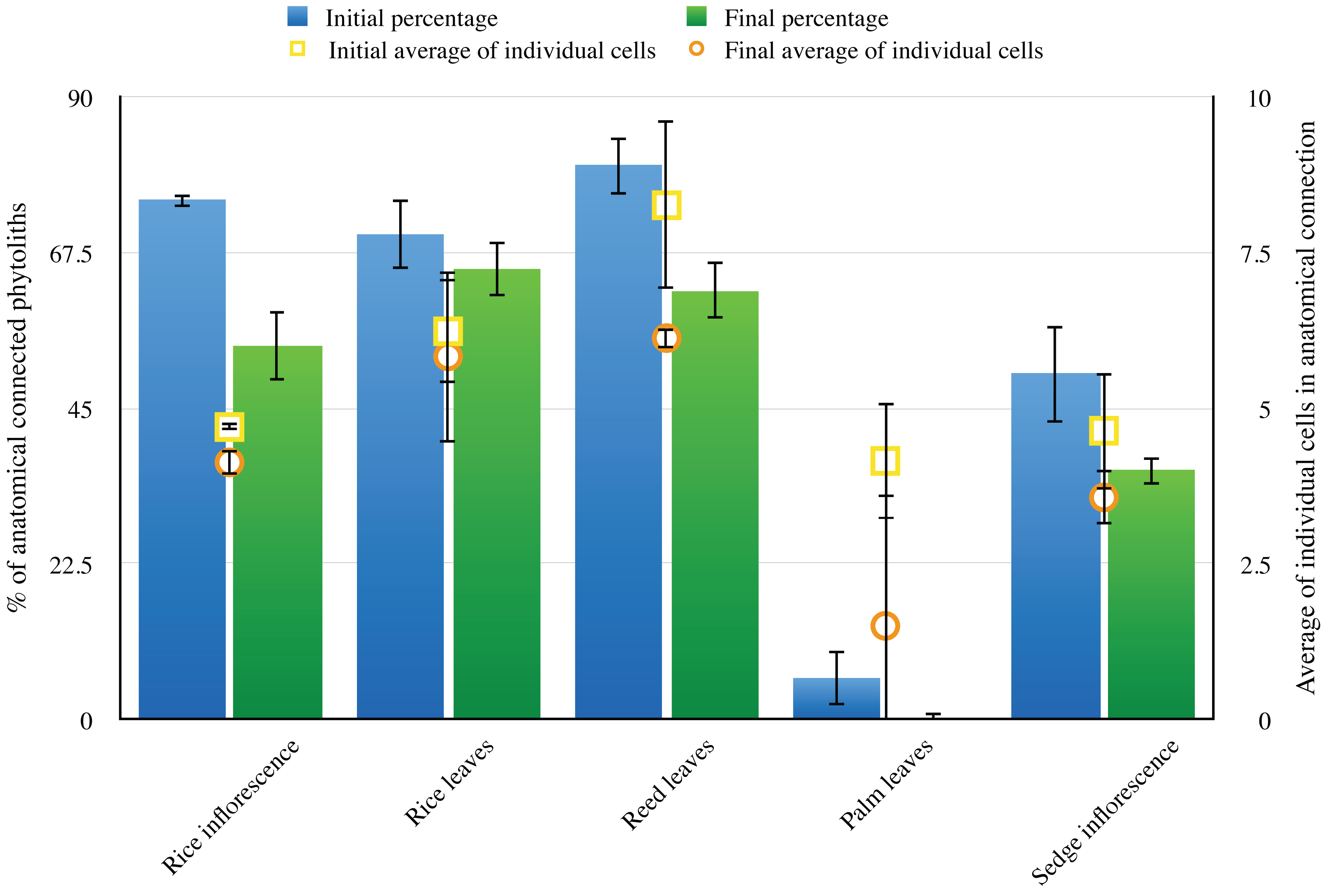

Supplement: S5 Fig — Light grey vertical bars: percentage of phytolith in anatomical connection before partial dissolution; dark gray vertical bars: percentage of phytoliths in anatomical connection after partial dissolution; circles: average number of phytoliths forming a conjoined multicellular structure before partial dissolution; squares: average number of phytoliths forming a conjoined multicellular structure after partial dissolution. Note the general reduction in the percentage of anatomically connected phytoliths, but the unchanged average amount of connected phytoliths per structure, following partial dissolution. (TIF) [file pone.0125532.s005.tif]
