# Supplementary material for: Understanding Fossil Phytolith Preservation: The Role of Partial Dissolution in Paleoecology and Archaeology
Source: PLoS One. 2015 May 20;10(5):e0125532. doi: 10.1371/journal.pone.0125532 (PMC4439089; doi:10.1371/journal.pone.0125532)
Supplement: S1 Table — Footnote: P.E. denotes elongated parallelepipedal phytoliths. (DOCX) [file pone.0125532.s006.docx]

| **Plant** | **Morphotype** | **Geometric shape used for calculation** | **N** | **Average surface area/volume** | **Maximum ratio** | **Minimum ratio** |
| --- | --- | --- | --- | --- | --- | --- |
| **Rice inflorescence** | Double peaked husk | Frustum of a cone | 10 | 1.26 ± 0.34 | 2.18 | 1.01 |
|  | P.E. psilate | Rectangular prism | 10 | 0.67 ± 0.22 | 1.09 | 0.35 |
|  |  |  |  |  |  |  |
| **Rice leaf** | Cuneiform bulliform | Irregular prism | 10 | 0.20 ± 0.04 | 0.26 | 0.13 |
|  | Bilobate short cell | Irregular prism | 12 | 0.95 ± 0.07 | 1.03 | 0.80 |
|  | P.E. psilate | Rectangular prism | 10 | 1.27 ± 0.19 | 1.64 | 0.90 |
|  | P.E. rugulate | Rectangular prism | 10 | 0.49 ± 0.17 | 0.81 | 0.28 |
|  |  |  |  |  |  |  |
| **Reed leaf** | Bilobate short cell | Irregular prism | 10 | 0.58 ± 0.12 | 0.79 | 0.42 |
|  | Rondel short cell | Frustum of a cone | 10 | 1.34 ± 0.11 | 1.11 | 1.26 |
|  | Cuneiform bulliform | Irregular prism | 10 | 0.12 ± 0.04 | 0.18 | 0.11 |
|  | Wavy long cell | Irregular prism | 13 | 0.94 ± 0.12 | 1.19 | 0.84 |
|  |  |  |  |  |  |  |
| **Palm leaf** | Spheroid/globular Echinate | Sphere | 26 | 0.78 ± 0.18 | 1.19 | 0.50 |
|  |  |  |  |  |  |  |
| **Sedge inflorescence** | Hat-shaped | Cone | 15 | 1.70 ± 0.23 | 2.41 | 1.45 |
|  | P.E. rugulate | Rectangular prism | 10 | 0.34 ± 0.06 | 0.45 | 0.24 |
