# Supplementary material for: Understanding Fossil Phytolith Preservation: The Role of Partial Dissolution in Paleoecology and Archaeology
Source: PLoS One. 2015 May 20;10(5):e0125532. doi: 10.1371/journal.pone.0125532 (PMC4439089; doi:10.1371/journal.pone.0125532)
Supplement: S2 Table — Measurements are expressed in μm, while surface area is expressed in μm2 and volume in μm3. P.E. denotes elongated parallelepipedal phytoliths. (DOCX) [file pone.0125532.s007.docx]

| **Rice inflorescence** | **Double peaked husk** | **Morphotype number** | **Radius 1** | **Radius 2** | **Height** | **Slant Height** | **Surface area** | **Volume** | **SA/V** |
| --- | --- | --- | --- | --- | --- | --- | --- | --- | --- |
|  | Frustum of a cone | 1 | 27.53 | 47.47 | 35.91 | 40.95 | 177538.77 | 162337.39 | 1.09 |
|  |  | 2 | 29.35 | 39.33 | 27.93 | 33.86 | 130345.98 | 104188.44 | 1.25 |
|  |  | 3 | 32.95 | 36.52 | 22.96 | 26.69 | 108464.55 | 87076.21 | 1.25 |
|  |  | 4 | 33.32 | 61.38 | 33.10 | 35.18 | 241348.65 | 239918.80 | 1.01 |
|  |  | 5 | 36.35 | 55.58 | 29.48 | 31.80 | 215719.60 | 198570.62 | 1.09 |
|  |  | 6 | 39.86 | 56.06 | 22.97 | 29.03 | 218649.17 | 167562.77 | 1.30 |
|  |  | 7 | 40.35 | 62.79 | 29.51 | 33.26 | 282228.05 | 250451.33 | 1.13 |
|  |  | 8 | 41.68 | 64.07 | 30.50 | 37.00 | 328736.73 | 271888.04 | 1.21 |
|  |  | 9 | 42.14 | 59.03 | 19.62 | 42.18 | 346183.27 | 159158.44 | 2.18 |
|  |  | 10 | 45.32 | 60.55 | 31.73 | 33.28 | 304857.55 | 281193.64 | 1.08 |
|  |  |  |  |  |  |  |  |  |  |
| **Rice inflorescence** | **P.E. psilate** | **Morphotype number** | **Length** | **Width** | **Thickness** |  | **Surface area** | **Volume** | **SA/V** |
|  | Rectangular prism | 1 | 25.67 | 4.54 | 3.53 |  | 446.05 | 410.81 | 1.09 |
|  |  | 2 | 34.67 | 18.53 | 3.75 |  | 1683.91 | 2409.18 | 0.70 |
|  |  | 3 | 36.36 | 6.18 | 3.75 |  | 768.34 | 842.47 | 0.91 |
|  |  | 4 | 43.32 | 14.96 | 4.46 |  | 1815.90 | 2889.46 | 0.63 |
|  |  | 5 | 58.84 | 8.70 | 7.82 |  | 2080.89 | 4005.67 | 0.52 |
|  |  | 6 | 60.76 | 10.84 | 5.91 |  | 2163.60 | 3893.02 | 0.56 |
|  |  | 7 | 70.98 | 16.03 | 6.06 |  | 3330.80 | 6897.12 | 0.48 |
|  |  | 8 | 75.17 | 23.67 | 8.26 |  | 5192.40 | 14704.66 | 0.35 |
|  |  | 9 | 76.55 | 8.67 | 3.82 |  | 1978.97 | 2536.73 | 0.78 |
|  |  | 10 | 99.25 | 5.23 | 6.47 |  | 2389.32 | 3356.40 | 0.71 |
|  |  |  |  |  |  |  |  |  |  |
| **Rice leaves** | **Bulliform cuneiform** | **Morphotype number** | **Area** | **Perimeter** | **Height** |  | **Surface area** | **Volume** | **SA/V** |
|  | Irregular prism | 1 | 594.57 | 105.96 | 23.67 |  | 3697.20 | 14073.50 | 0.26 |
|  |  | 2 | 690.03 | 107.53 | 24.54 |  | 4018.87 | 16932.65 | 0.24 |
|  |  | 3 | 796.08 | 121.14 | 33.61 |  | 5663.69 | 26756.20 | 0.21 |
|  |  | 4 | 889.39 | 134.02 | 45.57 |  | 7885.99 | 40529.34 | 0.19 |
|  |  | 5 | 1101.36 | 141.25 | 25.33 |  | 5780.28 | 27895.96 | 0.21 |
|  |  | 6 | 1137.50 | 146.17 | 32.16 |  | 6976.46 | 36586.95 | 0.19 |
|  |  | 7 | 1249.03 | 146.61 | 20.28 |  | 5471.28 | 25330.34 | 0.22 |
|  |  | 8 | 1402.05 | 163.53 | 20.92 |  | 6225.15 | 29330.85 | 0.21 |
|  |  | 9 | 1468.78 | 164.53 | 31.31 |  | 8088.93 | 45987.98 | 0.18 |
|  |  | 10 | 2274.71 | 196.27 | 44.12 |  | 13208.58 | 100356.48 | 0.13 |
|  |  |  |  |  |  |  |  |  |  |
| **Rice leaves** | **Bilobate short cell** | **Morphotype number** | **Area** | **Perimeter** | **Height** |  | **Surface area** | **Volume** | **SA/V** |
|  | Irregular prism | 1 | 51.00 | 34.20 | 7.72 |  | 366.18 | 393.89 | 0.93 |
|  |  | 2 | 53.76 | 36.76 | 5.84 |  | 322.33 | 314.15 | 1.03 |
|  |  | 3 | 62.16 | 39.35 | 6.03 |  | 361.46 | 374.61 | 0.96 |
|  |  | 4 | 62.76 | 40.04 | 5.79 |  | 357.25 | 363.23 | 0.98 |
|  |  | 5 | 62.99 | 41.34 | 6.48 |  | 393.90 | 408.24 | 0.96 |
|  |  | 6 | 66.45 | 42.60 | 5.35 |  | 361.01 | 355.79 | 1.01 |
|  |  | 7 | 72.04 | 36.88 | 7.05 |  | 404.06 | 507.86 | 0.80 |
|  |  | 8 | 74.18 | 44.93 | 4.99 |  | 372.77 | 370.49 | 1.01 |
|  |  | 9 | 83.13 | 47.57 | 6.60 |  | 480.12 | 548.42 | 0.88 |
|  |  | 10 | 83.77 | 53.74 | 5.58 |  | 467.49 | 467.52 | 1.00 |
|  |  | 11 | 83.90 | 50.52 | 5.92 |  | 466.69 | 496.35 | 0.94 |
|  |  | 12 | 95.92 | 49.52 | 5.70 |  | 474.10 | 546.75 | 0.87 |
|  |  |  |  |  |  |  |  |  |  |
| **Rice leaves** | **P.E. psilate** | **Morphotype number** | **Length** | **Width** | **Thickness** |  | **Surface area** | **Volume** | **SA/V** |
|  | Rectangular prism | 1 | 26.48 | 3.95 | 2.96 |  | 389.85 | 310.40 | 1.26 |
|  |  | 2 | 39.01 | 4.33 | 2.96 |  | 594.69 | 500.61 | 1.19 |
|  |  | 3 | 43.50 | 5.47 | 2.96 |  | 766.07 | 705.17 | 1.09 |
|  |  | 4 | 43.92 | 4.90 | 2.96 |  | 719.59 | 637.62 | 1.13 |
|  |  | 5 | 46.66 | 3.09 | 2.96 |  | 583.61 | 427.79 | 1.36 |
|  |  | 6 | 63.45 | 3.59 | 2.75 |  | 825.18 | 627.70 | 1.31 |
|  |  | 7 | 65.30 | 3.39 | 2.75 |  | 821.02 | 609.48 | 1.35 |
|  |  | 8 | 68.35 | 3.71 | 2.75 |  | 904.00 | 698.15 | 1.29 |
|  |  | 9 | 70.36 | 3.12 | 2.75 |  | 843.74 | 604.45 | 1.40 |
|  |  | 10 | 70.50 | 2.26 | 2.75 |  | 718.57 | 437.77 | 1.64 |
|  |  | 11 | 105.80 | 5.80 | 3.70 |  | 2052.40 | 2269.18 | 0.90 |
|  |  |  |  |  |  |  |  |  |  |
| **Reed leaves** | **Bilobate short cell** | **Morphotype number** | **Area** | **Perimeter** | **Height** |  | **Surface area** | **Volume** | **SA/V** |
|  | Irregular prism | 1 | 121.20 | 48.40 | 5.13 |  | 490.72 | 621.75 | 0.79 |
|  |  | 2 | 177.70 | 66.95 | 5.40 |  | 716.94 | 959.59 | 0.75 |
|  |  | 3 | 250.00 | 75.81 | 5.58 |  | 923.02 | 1394.99 | 0.66 |
|  |  | 4 | 294.04 | 83.54 | 6.11 |  | 1098.48 | 1796.59 | 0.61 |
|  |  | 5 | 326.89 | 84.63 | 8.48 |  | 1371.42 | 2772.06 | 0.49 |
|  |  | 6 | 355.95 | 94.32 | 8.70 |  | 1532.52 | 3096.73 | 0.49 |
|  |  | 7 | 404.38 | 98.71 | 7.94 |  | 1592.52 | 3210.82 | 0.50 |
|  |  | 8 | 416.89 | 103.11 | 6.12 |  | 1464.85 | 2551.40 | 0.57 |
|  |  | 9 | 433.67 | 102.86 | 7.19 |  | 1606.93 | 3118.09 | 0.52 |
|  |  | 10 | 549.39 | 116.76 | 9.71 |  | 2232.51 | 5334.57 | 0.42 |
|  |  |  |  |  |  |  |  |  |  |
| **Reed leaves** | **Rondel short cell** | **Morphotype number** | **Radius 1** | **Radius 2** | **Height** | **Slant Height** | **Surface area** | **Volume** | **SA/V** |
|  | Frustum of a cone | 1 | 3.82 | 7.33 | 8.08 | 7.88 | 908.83 | 815.43 | 1.11 |
|  |  | 2 | 6.05 | 8.55 | 5.54 | 5.98 | 1316.43 | 936.96 | 1.41 |
|  |  | 3 | 6.13 | 9.23 | 5.65 | 5.75 | 1408.24 | 1060.91 | 1.33 |
|  |  | 4 | 6.21 | 10.37 | 5.39 | 6.34 | 1741.15 | 1188.45 | 1.47 |
|  |  | 5 | 6.76 | 12.75 | 7.44 | 8.44 | 2939.02 | 2294.01 | 1.28 |
|  |  | 6 | 6.97 | 11.38 | 6.31 | 6.92 | 2282.31 | 1698.47 | 1.34 |
|  |  | 7 | 6.99 | 11.91 | 5.80 | 6.92 | 2408.94 | 1663.06 | 1.45 |
|  |  | 8 | 7.23 | 12.66 | 6.48 | 7.57 | 2845.72 | 2064.65 | 1.38 |
|  |  | 9 | 8.19 | 14.90 | 10.62 | 12.59 | 5734.04 | 4568.20 | 1.26 |
|  |  | 10 | 8.51 | 11.51 | 5.42 | 5.80 | 2428.23 | 1720.48 | 1.41 |
|  |  |  |  |  |  |  |  |  |  |
| **Reed leaves** | **Bulliform cuneiform** | **Morphotype number** | **Area** | **Perimeter** | **Height** |  | **Surface area** | **Volume** | **SA/V** |
|  | Irregular prism | 1 | 2967.89 | 263.72 | 29.05 |  | 13597.68 | 86227.52 | 0.16 |
|  |  | 2 | 3261.70 | 248.63 | 19.85 |  | 11459.42 | 64753.05 | 0.18 |
|  |  | 3 | 3849.76 | 272.31 | 47.91 |  | 20745.81 | 184441.81 | 0.11 |
|  |  | 4 | 4168.69 | 291.40 | 39.49 |  | 19845.88 | 164637.97 | 0.12 |
|  |  | 5 | 4168.69 | 291.40 | 31.04 |  | 17381.14 | 129377.85 | 0.13 |
|  |  | 6 | 4812.86 | 289.34 | 36.30 |  | 20127.63 | 174689.28 | 0.12 |
|  |  | 7 | 5719.13 | 312.89 | 46.27 |  | 25915.44 | 264618.58 | 0.10 |
|  |  | 8 | 6036.15 | 316.12 | 61.47 |  | 31503.42 | 371025.06 | 0.08 |
|  |  | 9 | 6314.04 | 348.06 | 38.36 |  | 25979.66 | 242205.24 | 0.11 |
|  |  | 10 | 6314.04 | 348.06 | 33.62 |  | 24330.36 | 212286.02 | 0.11 |
|  |  |  |  |  |  |  |  |  |  |
| **Reed leaves** | **Long cell wavy** | **Morphotype number** | **Area** | **Perimeter** | **Height** |  | **Surface area** | **Volume** | **SA/V** |
|  | Irregular prism | 1 | 351.14 | 117.90 | 3.11 |  | 1068.35 | 1090.29 | 0.98 |
|  |  | 2 | 368.02 | 113.38 | 3.26 |  | 1105.12 | 1198.04 | 0.92 |
|  |  | 3 | 455.46 | 153.37 | 3.26 |  | 1410.21 | 1482.69 | 0.95 |
|  |  | 4 | 525.04 | 173.55 | 3.11 |  | 1588.97 | 1630.26 | 0.97 |
|  |  | 5 | 544.35 | 123.70 | 3.26 |  | 1491.39 | 1772.06 | 0.84 |
|  |  | 6 | 550.14 | 149.44 | 3.26 |  | 1586.76 | 1790.91 | 0.89 |
|  |  | 7 | 771.32 | 204.08 | 3.26 |  | 2206.99 | 2510.93 | 0.88 |
|  |  | 8 | 779.67 | 219.53 | 3.26 |  | 2273.97 | 2538.11 | 0.90 |
|  |  | 9 | 810.27 | 258.69 | 3.67 |  | 2570.75 | 2976.28 | 0.86 |
|  |  | 10 | 814.53 | 203.21 | 2.16 |  | 2067.38 | 1756.93 | 1.18 |
|  |  | 11 | 840.56 | 250.15 | 3.67 |  | 2599.98 | 3087.55 | 0.84 |
|  |  | 12 | 995.03 | 264.90 | 2.16 |  | 2561.43 | 2146.25 | 1.19 |
|  |  | 13 | 1079.21 | 264.18 | 3.26 |  | 3018.43 | 3513.25 | 0.86 |
|  |  |  |  |  |  |  |  |  |  |
|  |  |  |  |  |  |  |  |  |  |
|  |  |  |  |  |  |  |  |  |  |
| **Palm leaf** | **Spheroid/Globular echinate** | **Morphotype number** | **Diameter** |  |  |  | **Surface area** | **Volume** | **SA/V** |
|  | Sphere | 1 | 5.045773 |  |  |  | 79.98 | 67.26 | 1.19 |
|  |  | 2 | 5.440169 |  |  |  | 92.98 | 84.30 | 1.10 |
|  |  | 3 | 5.688684 |  |  |  | 101.67 | 96.39 | 1.05 |
|  |  | 4 | 6.100857 |  |  |  | 116.93 | 118.90 | 0.98 |
|  |  | 5 | 6.212115 |  |  |  | 121.24 | 125.52 | 0.97 |
|  |  | 6 | 6.620984 |  |  |  | 137.72 | 151.97 | 0.91 |
|  |  | 7 | 7.059324 |  |  |  | 156.56 | 184.20 | 0.85 |
|  |  | 8 | 7.139422 |  |  |  | 160.13 | 190.54 | 0.84 |
|  |  | 9 | 7.141232 |  |  |  | 160.21 | 190.69 | 0.84 |
|  |  | 10 | 7.263252 |  |  |  | 165.73 | 200.63 | 0.83 |
|  |  | 11 | 7.397246 |  |  |  | 171.91 | 211.94 | 0.81 |
|  |  | 12 | 7.499617 |  |  |  | 176.70 | 220.86 | 0.80 |
|  |  | 13 | 7.535718 |  |  |  | 178.40 | 224.06 | 0.80 |
|  |  | 14 | 7.69356 |  |  |  | 185.95 | 238.44 | 0.78 |
|  |  | 15 | 7.798671 |  |  |  | 191.07 | 248.35 | 0.77 |
|  |  | 16 | 8.490512 |  |  |  | 226.47 | 320.48 | 0.71 |
|  |  | 17 | 8.785258 |  |  |  | 242.47 | 355.03 | 0.68 |
|  |  | 18 | 9.20339 |  |  |  | 266.10 | 408.17 | 0.65 |
|  |  | 19 | 9.353819 |  |  |  | 274.87 | 428.51 | 0.64 |
|  |  | 20 | 9.458242 |  |  |  | 281.04 | 443.03 | 0.63 |
|  |  | 21 | 9.787287 |  |  |  | 300.94 | 490.89 | 0.61 |
|  |  | 22 | 9.837337 |  |  |  | 304.02 | 498.46 | 0.61 |
|  |  | 23 | 10.03118 |  |  |  | 316.12 | 528.51 | 0.60 |
|  |  | 24 | 10.64498 |  |  |  | 355.99 | 631.59 | 0.56 |
|  |  | 25 | 11.43289 |  |  |  | 410.64 | 782.47 | 0.52 |
|  |  | 26 | 11.93182 |  |  |  | 447.26 | 889.44 | 0.50 |
|  |  |  |  |  |  |  |  |  |  |
| **Sedge inflorescence** | **Hat-shape** | **Morphotype number** | **Radius** | **Slant Height** | **Height** |  | **Surface area** | **Volume** | **SA/V** |
|  | Cone | 1 | 3.57 | 5.64 | 3.22 |  | 103.24 | 42.91 | 2.41 |
|  |  | 2 | 3.64 | 4.39 | 3.87 |  | 91.73 | 53.66 | 1.71 |
|  |  | 3 | 3.88 | 5.42 | 4.33 |  | 113.41 | 68.21 | 1.66 |
|  |  | 4 | 4.16 | 5.55 | 4.47 |  | 126.87 | 80.97 | 1.57 |
|  |  | 5 | 4.21 | 4.95 | 3.53 |  | 121.13 | 65.47 | 1.85 |
|  |  | 6 | 4.22 | 4.64 | 4.07 |  | 117.34 | 75.83 | 1.55 |
|  |  | 7 | 4.55 | 6.45 | 3.84 |  | 157.23 | 83.15 | 1.89 |
|  |  | 8 | 4.56 | 6.94 | 4.47 |  | 164.79 | 97.27 | 1.69 |
|  |  | 9 | 4.57 | 6.80 | 4.73 |  | 163.24 | 103.41 | 1.58 |
|  |  | 10 | 4.57 | 6.36 | 4.73 |  | 156.96 | 103.45 | 1.52 |
|  |  | 11 | 4.75 | 6.26 | 4.02 |  | 164.27 | 94.92 | 1.73 |
|  |  | 12 | 4.79 | 6.82 | 4.89 |  | 174.94 | 117.69 | 1.49 |
|  |  | 13 | 4.95 | 6.45 | 3.92 |  | 177.30 | 100.74 | 1.76 |
|  |  | 14 | 5.17 | 6.54 | 4.22 |  | 190.32 | 118.17 | 1.61 |
|  |  | 15 | 5.44 | 7.72 | 5.02 |  | 224.74 | 155.43 | 1.45 |
|  |  |  |  |  |  |  |  |  |  |
| **Sedge inflorescence** | **P.E. rugulate** | **Morphotype number** | **Length** | **Width** | **Thickness** |  | **Surface area** | **Volume** | **SA/V** |
|  | Rectangular prism | 1 | 57.95 | 13.95 | 11.20 |  | 3226.80 | 9050.88 | 0.36 |
|  |  | 2 | 78.02 | 10.79 | 8.49 |  | 3191.72 | 7147.76 | 0.45 |
|  |  | 3 | 68.65 | 18.08 | 15.10 |  | 5101.67 | 18743.13 | 0.27 |
|  |  | 4 | 59.39 | 16.71 | 8.37 |  | 3259.50 | 8311.08 | 0.39 |
|  |  | 5 | 98.55 | 11.67 | 11.23 |  | 4774.75 | 12911.11 | 0.37 |
|  |  | 6 | 93.84 | 18.42 | 14.06 |  | 6614.96 | 24310.22 | 0.27 |
|  |  | 7 | 100.31 | 19.11 | 11.49 |  | 6578.12 | 22026.21 | 0.30 |
|  |  | 8 | 67.26 | 17.61 | 13.35 |  | 4634.69 | 15810.13 | 0.29 |
|  |  | 9 | 55.04 | 15.24 | 13.68 |  | 3599.62 | 11469.57 | 0.31 |
|  |  | 10 | 57.52 | 19.80 | 18.96 |  | 5209.27 | 21589.85 | 0.24 |
